# Challenges and insights in Alagille syndrome: a case report

**DOI:** 10.1093/gastro/goae081

**Published:** 2024-08-22

**Authors:** Ricardo A Caravantes, Daniela Saenz, Juan P Cóbar, Zoe Kleiman

**Affiliations:** Department of Medical Research, Universidad Francisco Marroquín, Guatemala City, Guatemala; Department of Medical Research, Universidad Francisco Marroquín, Guatemala City, Guatemala; Department of Medical Research, Universidad Francisco Marroquín, Guatemala City, Guatemala; Department of Medical Research, Universidad Francisco Marroquín, Guatemala City, Guatemala

## Introduction

Alagille syndrome (ALGS) is a complex multisystemic disorder with a wide variety of clinical presentations. It is defined by a unique pattern of hepatic, cardiac, ophthalmic, skeletal, and facial anomalies and has an estimated prevalence of 1 in 30,000 to 1 in 70,000 live births [1]. Due to its variable presentation and overlapping clinical features with other genetic conditions, its diagnosis often proves elusive and requires a high index of suspicion [2].

The genetic component of ALGS has been well-established and consists of mutations in the Jagged1 (JAG1) or NOTCH2 genes. JAG1 and NOTCH2 genes play critical roles in embryonic development, particularly during hepatic, cardiac, ophthalmic, and skeletal organogenesis [3]. Genetic mutations disrupt the normal functioning of the Notch signaling pathway leading to the characteristic features of ALGS.

The hallmark hepatic manifestation of ALGS is cholestasis secondary to intrahepatic bile duct paucity, ultimately resulting in chronic liver disease [2]. Concurrently, cardiac anomalies, such as pulmonary artery stenosis and tetralogy of Fallot, contribute significantly to the morbidity and mortality associated with ALGS [2, 3].

In resource-limited settings, the diagnostic process can be particularly challenging due to limited access to advanced genetic testing and specialized imaging techniques. This case report emphasizes the diagnostic approach and collaboration required in such environments to reach an accurate diagnosis of ALGS.

The following case report describes a 3-month-old patient diagnosed with ALGS and details the clinical manifestations and diagnostic approach.

## Case report

A 3-month-old patient presented to the Emergency Department with a 1-month history of jaundice. The patient’s mother reported that a month following birth, the patient began to exhibit jaundice and acholic stools. Family medical history includes a father with liver disease of unknown etiology and hypercholesterolemia. Physical examination revealed full-body jaundice extending from the face to the lower extremities, palpable hepatosplenomegaly, and a cardiac murmur.

**Figure 1.**
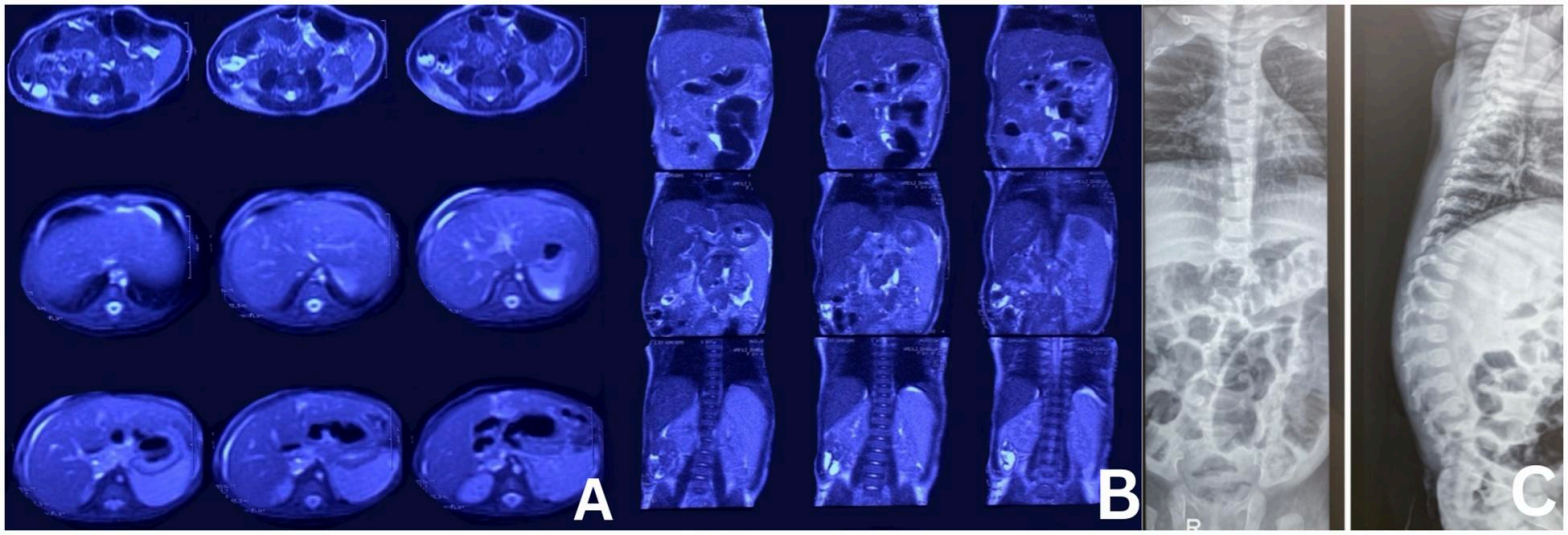
MRCP and X-ray images of the patient. (A) MRCP with evidence of hepatosplenomegaly and bile duct atresia (cross-sectional). (B) MRCP with evidence of hepatosplenomegaly and bile duct atresia (coronal plane). (C) Vertebral X-ray demonstrating the atypical butterfly wings pattern.

Initial laboratory testing revealed leukocytosis and hypercholesterolemia. An upper abdominal ultrasound showed cholelithiasis. Due to these findings, a magnetic resonance cholangiopancreatography (MRCP) was performed. MRCP reported hepatomegaly with fatty changes, splenomegaly, and bile duct atresia (Figure 1A and B). Additional laboratory studies demonstrated elevated alkaline phosphatase, transaminases, bilirubin, gamma-glutamyl transferase, and ammonia. As part of the initial evaluation, screening for infectious disease was conducted and revealed positive IgM titers for cytomegalovirus, which was treated with ganciclovir without clinical or laboratory improvement.

Considering the radiologic evidence of a hepatobiliary disease process, the decision to perform a 99mTc-GSA SPECT/CT was made. Nuclear imaging revealed a normal liver without gallbladder filling and no evident contrast emptying into the biliary system at the time of the study or 24 h later. Chloride sweat test was performed due to suspected cystic fibrosis, which resulted in inconclusive test. Due to suspicion of cystic fibrosis, a genetic test was performed, which gave ALGS results.

Since the results of the genetic test were relevant for ALGS, additional studies were performed. Vertebral X-rays showed butterfly wing vertebrae (Figure 1C) and an ophthalmological examination revealed the presence of posterior embryotoxon. Likewise, a liver biopsy was performed, which showed signs of cirrhosis and obliterative cholangiopathy. Acyanotic congenital heart disease was diagnosed through an echocardiogram, which demonstrated a persistent foramen ovale of 2.5 mm and a patent ductus arteriosus of 2 × 1.8 mm.

Post diagnosis, the patient was managed with a multidisciplinary approach involving hepatology, cardiology, and genetic counseling. Treatment was given to manage cholestasis and pruritus, and regular follow-up was established for monitoring liver function and cardiac status. Genetic counseling was provided to the family to discuss the implications of the diagnosis and future reproductive options. An appointment was scheduled to evaluate the possibility of a liver transplant; however, follow-up was lost.

## Discussion

ALGS is a rare autosomal dominant disorder characterized by various clinical features predominantly affecting the liver, heart, vertebrae, and face [4]. First described by Daniel Alagille in 1969, ALGS has since been recognized as a multisystemic disorder with significantly variable clinical presentation and severity [4, 5]; 40.3% of children reach adulthood with their native liver, and have an increased risk of hepatic and cardiovascular complications during adulthood [2, 6]. Given that liver involvement due to intrahepatic bile duct paucity and cholestasis is a hallmark of ALGS, chronic liver disease can sometimes develop, progressing to cirrhosis and liver failure.

Cardiovascular anomalies are prevalent and usually involve the pulmonary arteries and cardiac valves. Common abnormalities include pulmonary artery stenosis, tetralogy of Fallot, and ventricular septal defects. Untreated congenital heart defects may persist and cause pulmonary hypertension and heart failure. Butterfly vertebrae, posterior embryotoxon, and characteristic facial features such as a prominent forehead, deep-set eyes, and a pointed chin, contribute to the clinical phenotype of ALGS [7]. This condition is classified as complete when four or more organ systems are involved; otherwise, it is considered incomplete [8].

ALGS is primarily caused by mutations in the JAG1 or the NOTCH2 genes. These mutations disrupt normal cellular signaling pathways involved in organogenesis, therefore the hallmark biliary atresia [9].

Diagnosing ALGS involves integrating clinical observations, medical history, physical examination, and specific medical tests. Physicians assess for signs or symptoms across various organ systems including the liver, heart, eyes, facial features, skeleton, blood vessels, and kidneys. A diagnosis may be confirmed if a patient exhibits manifestations in three or more of these areas [9]. Alternatively, if signs are present in two areas and there is a family history of ALGS, diagnosis may also be considered [10]. Clinical suspicion should be raised in infants with cholestatic jaundice and characteristic facies. Imaging modalities, such as ultrasonography and MRCP, may be used to assess hepatobiliary anatomy [11]. Echocardiography is essential to determine the presence of cardiac abnormalities. Genetic testing is the gold standard for diagnostic confirmation [11].

The management of ALGS is aimed at alleviating symptoms, preventative care, and optimization of long-term outcomes. Persistent liver involvement is managed with supportive care and medication to mitigate cholestasis and pruritus. In cases of end-stage liver disease, transplantation may be the only viable treatment option [12]. Cardiac abnormalities should be monitored frequently, and operative therapy should be considered in the appropriate setting. Finally, regular follow-up evaluations with liver function tests, echocardiography, and developmental assessments are imperative to monitor disease progression [12].

## Conclusions

ALGS is a rare genetic disorder characterized by liver, heart, eye, and skeletal abnormalities due to mutations in JAG1 or NOTCH2 genes. This case report underscores the complex clinical scenario and comprehensive evaluation associated with ALGS. Advances in genetic testing have facilitated early diagnosis, allowing for interventions aimed at optimizing patient outcomes. Long-term follow-up is essential to monitor disease progression, prevent complications, and address evolving medical needs. This report also highlights the unique challenges and innovative strategies required to diagnose and manage ALGS in resource-limited settings, emphasizing the importance of increased awareness and collaboration among healthcare professionals in recognizing and managing rare genetic disorders like ALGS.

## Authors’ Contributions

R.A.C., D.S., and Z.K collected the data and drafted the manuscript. J.P.C. revised the manuscript. R.A.C. and D.S. worked on the final approval of the version to be published. R.A.C., D.S., J.P.C. and Z.K. read and approved the final version of the manuscript.
